# Evaluating Pre-Immobilization and Post-Immobilization Bioimprinting Strategies for the Activation of Lipases: A Case Study of LipC12

**DOI:** 10.17113/ftb.63.03.25.8940

**Published:** 2025-09-14

**Authors:** Leonardo Pellizzari Wielewski, Maria Lujan Ferreira, Robson Carlos Alnoch, David Alexander Mitchell, Nadia Krieger

**Affiliations:** 1 Postgraduate Program in Science - Biochemistry, Federal University of Paraná, P.O. Box 19046, Polytechnic Center, Curitiba 81531-980, Paraná, Brazil; 2 Department of Chemistry, National University of the South (UNS), Avda Alem 1253, Bahía Blanca 8000, Argentina; 3 Pilot Plant of Chemical Engineering - PLAPIQUI (UNS-CONICET), Bahía Blanca 8000, Argentina; 4 Department of Biology, Faculty of Philosophy, Sciences and Letters of Ribeirão Preto, University of São Paulo, Ribeirão Preto, São Paulo, 14040-901, Brazil; 5 Department of Biochemistry and Molecular Biology, Federal University of Paraná, P.O. Box 19046, Polytechnic Center, Curitiba 81531-980, Paraná, Brazil; 6 Department of Chemistry, Federal University of Paraná, P.O. Box 19032, Polytechnic Center, Curitiba 81531-980, Paraná, Brazil

**Keywords:** bioimprinting, lipases, immobilization, activation, enhancement of enzymatic properties

## Abstract

**Research background:**

Although there are many studies of the bioimprinting of lipases, there is no study comparing the strategies of bioimprinting prior to immobilization (pre-immobilization) and bioimprinting after immobilization (post-immobilization). Likewise, there is no study that compares bioimprinting of lipases immobilized from a pure lipase preparation and lipases immobilized from a crude extract. We therefore investigated these strategies, using the metagenomic lipase LipC12.

**Experimental approach:**

We immobilized LipC12 covalently on the commercial support Immobead 150P and treated it with various bioimprinting agents, either pre-immobilization or post-immobilization. We also compared immobilization from a pure LipC12 preparation and immobilization from a crude cell-free extract.

**Results and conclusions:**

The best improvements in triolein-hydrolyzing activity in *n*-hexane, compared to a non-bioimprinted control, were obtained with post-immobilization bioimprinting, using oleic acid dissolved in *t*-butanol: a 12-fold improvement for immobilization from a pure LipC12 preparation and an almost 14-fold improvement for immobilization from the crude cell-free extract. This bioimprinting agent also gave a 3.5-fold increase in activity for the synthesis of ethyl oleate in *n*-hexane, this result being obtained for pre-immobilization bioimprinting and immobilization from the cell-free extract.

**Novelty and scientific contribution:**

This study is the first to compare pre-immobilization and post-immobilization bioimprinting strategies, as well as bioimprinting of enzymes immobilized from both pure enzyme preparations and crude cell-free extracts. These results encourage further investigation into bioimprinting strategies.

## INTRODUCTION

Immobilized lipases (EC 3.1.1.3) are widely used in industries, as immobilization allows for the reuse of the enzyme in multiple reaction cycles and overcomes limitations associated with free enzymes. Immobilization simplifies handling, prevents product contamination, enhances stability, increases catalyst productivity, and improves cost-effectiveness, while also facilitating the use of fixed-bed bioreactors and the intensification of industrial processes (*1*–*3*). However, water-restricted media are often used to favor synthetic reactions in industrial applications and enzyme activities are often low and stability is often poor in these media, even when the enzyme is immobilized (*1*, *4*–*6*). Addressing these challenges is crucial for expanding lipase applications.

One approach to enhancing the activity of immobilized lipases in water-restricted media is bioimprinting, also known as molecular imprinting. In this technique, lipases are treated with specific compounds known as bioimprinting agents or templates (*7*–*11*). These agents create distinct binding or recognition sites for target molecules within the lipase structure, modifying it and activating the enzyme. Upon the removal of the bioimprinting agent, the enzyme retains its activated structure (*12*–*14*). Bioimprinting is typically done using substrates, products, or their analogs. Other compounds that positively interact with lipases, such as surfactants (*15*), solvents (*6*), and polymers like polyethylene glycol (PEG) (*12*), have also been utilized as bioimprinting agents, although they are not always explicitly labeled as such and are frequently categorized as pretreatment agents. In this work, we adopt a broad definition of bioimprinting agents, encompassing compounds that interact with lipases to enhance their activity, including solvents and surfactants.

It has been suggested that bioimprinting increases lipase activity by promoting the opening of the lid domain that typically covers the active site (*7*, *10*, *16*, *17*), mirroring the natural activation of lipases at interfaces. It has also been suggested that the bioimprinting agent promotes a conformation resembling that of the enzyme-substrate complex and that this conformation is maintained after the bioimprinting agent is removed, even in the absence of the substrate (*12*, *18*, *19*).

Bioimprinting of lipases, coupled with immobilization, has been well investigated to enhance activity and stability in hydrolysis and esterification reactions (*7*, *20*–*27*), and to improve enantioselectivity (*23*, *24*). The immobilization methods used in these studies include adsorption (*26*, *27*) and covalent immobilization (*3*, *25*). Most research has been done with commercial enzymes, such as lipases from *Candida rugosa* (CRL) (*7*), *Burkholderia cepacia* (BCL) (*20*) and lipase B of *Candida antarctica* (CALB) (*21*).

Across all these studies cited above, the bioimprinting agents are added either prior to immobilization or during the immobilization step itself, there being no study that compares bioimprinting prior to immobilization and bioimprinting after immobilization. Likewise, there is no study that compares bioimprinting of lipases immobilized from a pure lipase preparation and lipases immobilized from a crude extract.

In the current study, we explore these strategies using the recombinant metagenomic lipase LipC12 and Immobead 150P as the support for LipC12 immobilization. This work extends the previous studies of bioimprinting of LipC12 immobilized on Immobead 150P done by Sanchez *et al*. (*25*): they only tested the addition of the bioimprinting agent, oleic acid dissolved in *t*-butanol, prior to immobilization. Additionally, they only tested LipC12 immobilized from a purified fraction, they did not test the effect of bioimprinting on LipC12 immobilized from the crude extract. We assessed the performance of the combined bioimprinting and immobilization strategies based on hydrolytic activity (hydrolysis of triolein) and synthetic activity (esterification of oleic acid with ethanol), both evaluated in organic media (*n*-hexane). Additionally, we analyzed the reuse of bioimprinted immobilized LipC12 in successive cycles for both hydrolytic and esterification reactions.

## MATERIALS AND METHODS

### Materials

Luria Bertani (LB) and Luria-Agar (LA) culture media were used, with the latter prepared by incorporating agar (15 g/L) (Conda Laboratories SA, Madrid, Spain) into LB medium. Isopropyl-β-d-thiogalactopyranoside (IPTG; Invitrogen Life Technologies, Carlsbad, CA, USA) was used to induce lipase expression. Affinity columns (HiTrap Chelating HP; GE Healthcare, Uppsala, Sweden) were used to purify LipC12. The *Escherichia coli* TOP10 strain (Invitrogen Life Technologies) was used for plasmid storage at -80 °C and the BL21(DE3) strain (Novagen, Madison, WI, USA) was used for expression. The immobilization support was Immobead 150P (Sigma-Aldrich, Merck, St. Louis, MO, USA), with a particle size of 0.15–0.5 mm. Triethylamine, *n*-heptane, *n*-hexane (99.5 %), *t*-butanol and toluene were from Vetec (Duque de Caxias, RJ, Brazil), ethanol (99.5 %), glycerin, hydrochloric acid, acetic acid, isopropanol and Tween 80 were from Synth (Diadema, SP, Brazil), tris(hydroxymethyl)aminomethane and imidazole were from Neon Química (Suzano, SP, Brazil). Cetyltrimethylammonium bromide (CTAB), Coomassie R-250, triolein (65 %), oleic acid (90 %) and kanamycin were from Sigma-Aldrich, Merck. Commercial olive oil (Gallo brand) was bought at a local supermarket. All other reagents, such as salts used for solution preparation, surfactants and reaction substrates, were of analytical grade.

### Overexpression and purification of LipC12

LipC12 was produced and purified according to Glogauer *et al*. (*28*), with slight modifications. *E. coli* BL21(DE3), carrying the plasmid pET28a-lipC12, was cultivated at 37 °C in 800 mL of LB medium containing kanamycin (50 μg/mL), in 2-liter Erlenmeyer flasks. When the absorbance at *λ*=600 nm reached value of 0.600, IPTG was added (to give a concentration of 0.5 mmol/L) for induction and the culture was incubated for another 16 h, at 20 °C. The broth was centrifuged (CR21E; Hitachi, Tokyo, Japan) (4000×*g*) at 18 °C for 10 min, giving a cell pellet that was then resuspended in 50 mL of lysis buffer (50 mmol/L Tris-HCl, pH=7.5, 500 mmol/L NaCl, 10 mmol/L imidazole) and sonicated in an ice bath using a SONICATOR® XL 2020 (Heat Systems-Ultrasonics Inc., New York, NY, USA; twelve 20-second pulses of 90 W, with 30-second intervals). Cell debris was removed by centrifugation (15 000×*g*) of the crude extract at 4 °C for 15 min. The supernatant was purified with a HiTrap column, previously loaded with Ni^2+^ and equilibrated with lysis buffer. After loading with the His-tagged protein, the column was eluted stepwise, with increasing concentrations (50 to 500 mmol/L) of imidazole in a buffer containing 50 mmol/L Tris-HCl (pH=7.5) and 500 mmol/L NaCl. Two column volumes of buffer were passed at each imidazole concentration. The fractions that contained proteins were analyzed by SDS-PAGE and then pooled and dialyzed with 50 mmol/L Tris-HCl buffer (pH=7.5) containing 150 mmol/L NaCl and 10 mmol/L CaCl_2_.

The purified LipC12 preparation had a protein concentration of 2.3 mg/mL, determined by the bicinchoninic acid (BCA) method (*29*) using a kit (Pierce Biotechnology, Rockford, IL, USA), and had a specific olive-oil-hydrolyzing activity of (1948±88) U/mg (mean±standard deviation, *N*=5). The cell-free crude extract of LipC12 had a specific olive-oil-hydrolyzing activity of (2774±139) U/mg (*N*=5) and a total protein concentration of 14.5 mg/mL. Densitometry analysis of an SDS-PAGE gel showed that LipC12 represented 29.4 % of the total protein (*i.e.* 4.26 mg/mL). A 10-µL aliquot of 0.01 % (*m*/*V*) sodium azide was added to the enzyme solution, which was then stored at 4 °C. The specific activity of this LipC12 solution remained constant during the studies.

### Standard procedure for immobilization of LipC12

LipC12 was immobilized through covalent binding on Immobead 150P. Two different immobilization solutions were used: *i*) crude cell-free extract that had been centrifuged to eliminate cell debris, and *ii*) a solution of purified enzyme. The optimized protocol of Madalozzo *et al*. (*30*) was used, with minor adaptations.

Dry Immobead 150P beads were used, without pretreatment. A mass of 0.1 g of support was added to 5 mL of LipC12 solution at pH=7.5. This represented a protein loading of 10 mg/g for the purified preparation and a protein loading of 200 mg/g for the crude extract. The suspension was incubated on an orbital shaker (TE-421; TECNAL, Piracicaba, SP, Brazil) at 150 rpm for 6 h at 4 °C. The olive-oil-hydrolyzing activity of the lipase-containing supernatant offered for immobilization was followed during this incubation. After the incubation, the immobilized preparation was washed (3×) with 50 mL of 50 mmol/L Tris-HCl buffer (pH=7.5). It was then recovered by filtration through qualitative filter paper (Whatman n° 15), desiccated for 16 h under partial vacuum at 4 °C and stored at -20 °C.

The immobilization efficiency (IE/%) achieved at the end of the 6-hour incubation was calculated as follows:


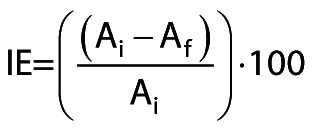
 /1/

where A_i_ is the olive-oil-hydrolyzing activity (U) of the supernatant before the addition of Immobead 150P and A_f_ is the olive-oil-hydrolyzing activity (U) that remains in the supernatant after the incubation.

The activity retention (AR/%) was calculated as follows:


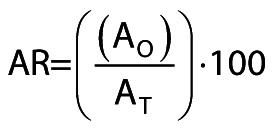
 /2/

In this equation, A_O_ represents the triolein-hydrolyzing activity of the immobilized preparation (which was measured in *n*-hexane) and A_T_ represents the theoretical olive-oil-hydrolyzing activity of the immobilized preparation. This theoretical value was calculated as follows:


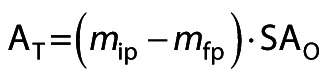
 /3/

where *m*_ip_ and *m*_fp_ are the masses of protein in the supernatant before and after immobilization, respectively. SA_O_ is the specific triolein-oil-hydrolyzing activity (U/mg of protein), measured in *n*-hexane, of the free enzyme. If the lipase is activated upon immobilization, then AR values can be above 100 %.

Control experiments were done with the enzyme solution being incubated under the immobilization conditions for 24 h, but without the supports. The supernatants in these control experiments showed no loss of enzymatic activity.

To confirm that LipC12 was covalently immobilized on Immobead 150P, desorption studies were done. Immobilized LipC12 (20 mg) was placed in Eppendorf tubes that contained 2 mL of a 2 % (*m*/*V*) sodium dodecyl sulfate (SDS) solution in distilled water. After a 30-minute incubation in boiling water (*31*), the immobilized enzyme was removed by filtration, and the protein content in the filtrate was determined using the BCA method (*29*). No protein was detected in the filtrate of LipC12 immobilized from the purified fraction, whereas LipC12 immobilized from the cell-free crude extract gave a low concentration of protein of 0.06 mg/mL, below the lower level of sensitivity of the method. These results show that LipC12 was effectively attached to the support through covalent bonding.

### Bioimprinting strategies

Two bioimprinting strategies were used with LipC12 immobilized onto Immobead 150P, using either purified LipC12 or the cell-free crude extract containing LipC12: (*i*) pre-immobilization, with the bioimprinting agents added to the solution of free LipC12 prior to immobilization, and (*ii*) post-immobilization, with the bioimprinting agents added to immobilized LipC12.

Pre-immobilization bioimprinting was done in sealed 25-mL Erlenmeyer flasks, each containing enzyme solution (either 3 or 4 mL; independently of the volume, the solution contained 1 mg of purified LipC12 or, in the case that the crude extract was used for immobilization, 20 mg of protein) and 1 or 2 mL of the bioimprinting agents (Table 1). The flask was incubated at 4 °C on an orbital shaker (TE-421; TECNAL) at 150 rpm, initially for 15 min, and then for a further 6 h after the addition of 0.1 g of Immobead 150P. The immobilization efficiency (IE, Eq. 1) and retention of activity (AR, Eq. 2) were then determined, based on the olive-oil-hydrolyzing activity, measured in aqueous medium. The immobilized derivative was then washed with 5 mL of 50 mmol/L Tris-HCl buffer, pH=7.5, filtered under vacuum (through Whatman filter paper n° 15), and dried for 24 h at 4 °C in a vacuum desiccator. After drying, it was washed twice more with *t*-butanol (5 mL each time) and dried once more for 24 h under vacuum in the desiccator. Finally, it was stored in Eppendorf tubes at -20 °C until use.

**Table 1:** Solutions used for pre and post-immobilization bioimprinting

Acronym	*V*(OA)/µL	*n*(OA)/nmol)*	Bioimprinting agent		*V*(purified LipC12 solution or crude extract)***/µL
			*V*(other solvent)/µL	*V*(CTAB)_aq_**/µL	
OA1	58.82	29.4	*t*-BUT, 941.18	-	4000
OA5	291.1	147	*t*-BUT, 705.9	-	4000
CTAB	-	-	-	1000	4000
CTAB-*t*-butanol	-	-	*t*-BUT, 1000	1000	3000
CTAB-OA1	58.82	29.4	*t*-BUT, 941.18	1000	3000
CTAB-OA5	291.1	147	*t*-BUT, 705.9	1000	3000
MET	-	-	MET, 1000	-	4000
ETH	-	-	ETH, 1000	-	4000
BUT	-	-	*t*-BUT, 1000	-	4000
HEP	-	-	*n*-HEP, 1000	-	4000
TOL	*-*	*-*	TOL, 1000	-	4000

*Oleic acid (OA) was dissolved in *t*-butanol before being added to the enzyme solution. Higher amounts of oleic acid were not utilized since they inhibit LipC12 activity (data not shown). **c(CTAB)=50 mmol/L. ***Regardless of the volume, this enzyme solution contained either 1 mg of purified LipC12 (when purified enzyme was used) or 20 mg protein (when the crude extract was used). Since 0.1 g Immobead 150P was added after the bioimprinting, the protein loading in the immobilization was 10 mg per g of support when pure LipC12 was used and 200 mg of protein per g of support when crude extract was used. CTAB=cetyltrimethylammonium bromide, MET=methanol, ETH=ethanol, BUT=butanol, HEP=heptane and TOL=toluene

Post-immobilization bioimprinting was done with 0.1 g of dried immobilized LipC12 preparation (obtained using the standard LipC12 immobilization procedure described above). This preparation was added to a sealed 25-mL Erlenmeyer flask with 4 mL of Tris-HCl buffer (50 mmol/L, pH=7.5) and 1 mL of the bioimprinting solution (Table 1). The flask was incubated on a shaker (150 rpm) at 4 °C for 15 min. The immobilized derivative was then recovered by filtration (Whatman filter paper n° 15) under vacuum. After being washed twice with *t*-butanol (5 mL each time) to remove the bioimprinting agents, it was dried for 24 h at 4 °C in a vacuum desiccator and then stored in Eppendorf tubes at -20 °C until use.

### Reuse of bioimprinted immobilized LipC12

Two bioimprinted immobilized derivatives, prepared through immobilization of LipC12 from a crude extract, were reused over multiple cycles of triolein hydrolysis in *n*-hexane: one derivative underwent pre-immobilization bioimprinting with 147 nmol oleic acid (OA5), while the other underwent post-immobilization bioimprinting with 29.4 nmol oleic acid (OA1). After each cycle, the immobilized derivatives were recovered by vacuum filtration (Whatman filter paper n° 15) and washed twice with *n*-hexane (5 mL each time). They were then dried at 4 °C for 16 h in a vacuum desiccator and added to fresh reaction medium for the next cycle. The activities are reported as percentages of the absolute conversion that was achieved in the first cycle.

These tests were done with only *φ*(water)=2 % added to the reaction medium, as bioimprinting tends to be ineffective at high water volume fractions, which allow the lipase molecule sufficient flexibility for it to revert to the structure that it had prior to the bioimprinting (*22*).

### Analytical methods

#### Determination of olive-oil-hydrolyzing activity in aqueous medium

The purification and immobilization of LipC12 were monitored based on the olive-oil-hydrolyzing activity at 37 °C in aqueous medium, determined with an automatic titrator pHStat (Metrohm 718 Stat Titrino). The reaction mixture (20 mL) contained 3 % (*m*/*V*) gum arabic, 2 mmol/L CaCl_2_, 2.5 mmol/L Tris-HCl buffer (pH=7.5), 150 mmol/L NaCl and 67 mmol/L of olive oil, emulsified in distilled water. The enzyme solution was added to the emulsion, with magnetic stirring at 300 rpm. The reaction was monitored for 5 min (*32*). One unit (U) of olive-oil-hydrolyzing activity in aqueous medium was defined as the release of 1 μmol of fatty acid per minute, under the assay conditions.

#### Determination of triolein-hydrolyzing activity in *n*-hexane

The triolein-hydrolyzing activities of bioimprinted immobilized preparations were determined in *n*-hexane. An amount of 70 mmol of triolein, 0.1 mL of distilled water and 20 mg of immobilized lipase were added to 4.6 mL of *n*-hexane in a 125-mL Erlenmeyer flask, which was then incubated on an orbital shaker (TE-421; TECNAL) at 200 rpm and 40 °C. The oleic acid concentration was determined by the method of Lowry and Tinsley (*33*). The reaction was followed for 25 min, with samples taken every 5 min, with these data being used to determine the initial reaction rate. One unit (U) of triolein-hydrolyzing activity in *n*-hexane was defined as the production of 1 µmol of oleic acid per minute, under the assay conditions.

The relative hydrolytic activities (R_H_, %) of the bioimprinted immobilized preparations were calculated as follows:


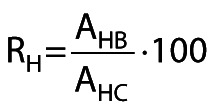
 /4/

where A_HB_ is the triolein-hydrolyzing activity (U/g of support) of the bioimprinted preparation and A_HC_ is the triolein-hydrolyzing activity (U/g of support) of the control preparation (*i.e.* the corresponding test with immobilized LipC12 that was not bioimprinted).

#### Determination of esterification activity in *n*-hexane

The best bioimprinted immobilized preparations were also evaluated based on their esterification activity (synthesis of ethyl oleate), using a slight modification of the method of Madalozzo *et al*. (*30*). The reaction medium (5 mL) contained *n*-hexane, oleic acid (70 mmol/L) and ethanol (210 mmol/L). It was prepared in 25-mL Erlenmeyer flasks, which were incubated in an orbital shaker (TE-421; TECNAL) at 180 rpm and 40 °C. Immobilized preparation (either 50 or 110 mg) was added to start the reaction. Samples (100 μL) were collected every 5 min during 60 min and their free fatty acid contents were determined by the Lowry and Tinsley method (*33*), using calibration curves obtained with oleic acid. The initial reaction rate was calculated. One unit (U) of esterification activity in *n*-hexane was defined as the disappearance of 1 µmol of fatty acid per minute, under the assay conditions. There was no reaction in a control flask prepared identically, except that enzyme was not added.

The relative esterification activities (R_E_, %) of the bioimprinted immobilized preparations were calculated as follows:


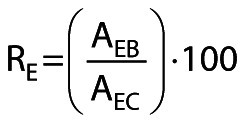
 /5/

where A_EB_ is the esterification activity (U/g of support) of the bioimprinted preparation and A_EC_ is the esterification activity (U/g of support) of the control preparation (*i.e.* the corresponding test with immobilized enzyme that was not bioimprinted).

#### SDS-PAGE and densitometry

**Fig. S1:**
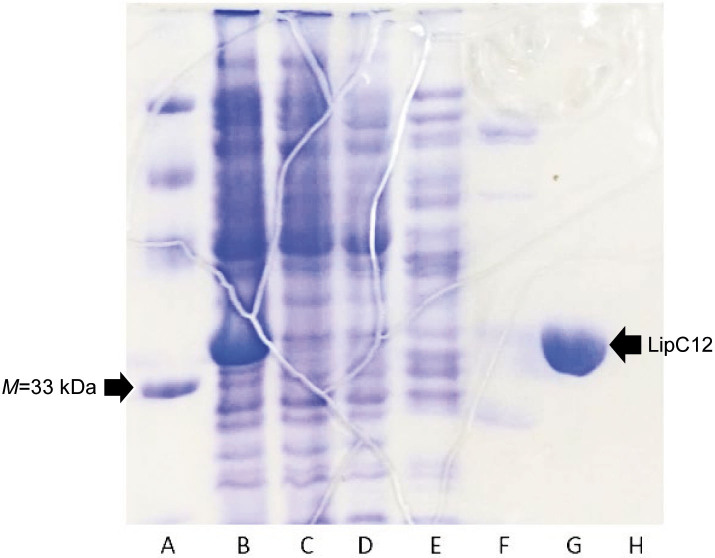
Electrophoresis gel (SDS-PAGE) showing the crude extract and purified fractions of LipC12. Legend: A=molecular mass markers, LipC12=metagenomic lipase, B=crude extract, C=elution from the application of the crude extract to the column. Fractions eluted with imidazole (concentration gradient in mmol/L): D=10, E=50, F=100, G=300 and H=500

The crude extract and purified fractions of LipC12 were analyzed through SDS-PAGE (*34*). A stacking gel with 5 % (*m*/*V*) polyacrylamide and a separating gel with 12 % (*m*/*V*) polyacrylamide were used. The samples were heated at 100 °C for 5 min before application. The proteins within the gel were stained for 30 min with 0.05 % (*m*/*V*) Coomassie Brilliant Blue R-250. Destaining was done for 60 min with a mixture of *V*(methanol):*V*(acetic acid):*V*(water)=5:1:4. The molecular mass markers were α-lactalbumin (14.4 kDa), trypsin inhibitor (20.1 kDa), carbonic anhydrase (31 kDa), ovalbumin (45 kDa), bovine serum albumin (BSA) (66 kDa) and phosphorylase b (97 kDa) (Pierce Biotechnology). The runs were conducted for at least 60 min at a constant voltage of 150 V (Fig. S1).

**Fig. S2:**
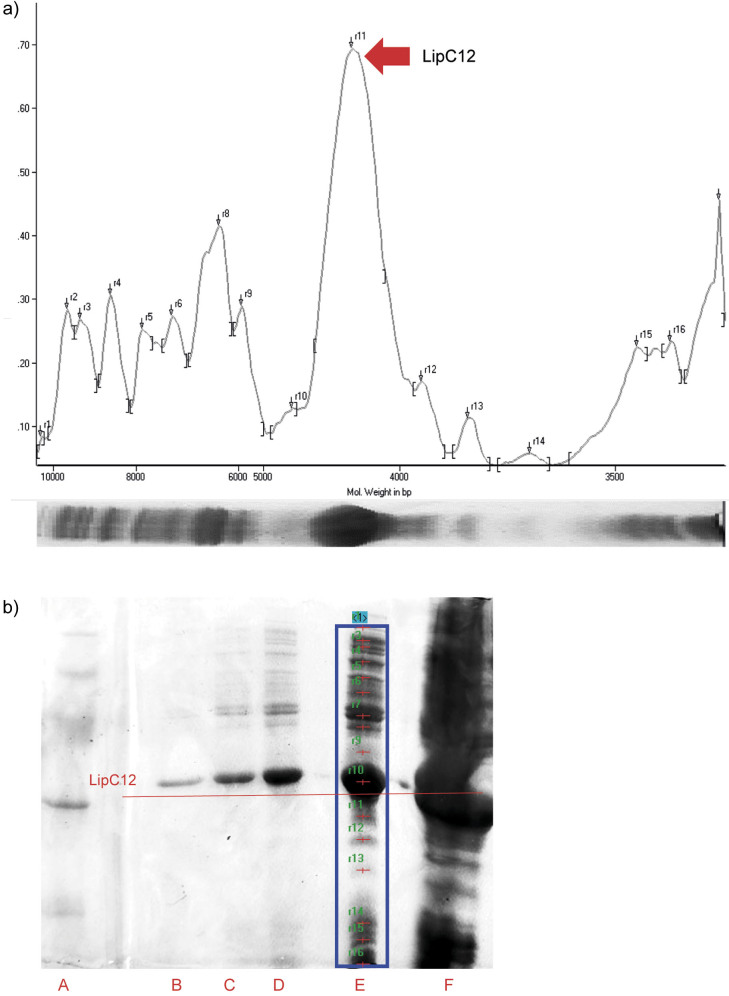
Electrophoresis gel (SDS-PAGE) and densitometry analysis of the gel for LipC12: a) image densitometry containing the dilutions of the crude extract, and b) absorbance of each detected band. Legend: A=molecular mass markers and dilutions of the LipC12 crude extract: B=200×, C=100×, D=50×, E=10×, F=1×

To determine the relative concentration of LipC12 in the cell-free crude extract, the SDS-PAGE gel was analyzed by densitometry using LabWorks Image Acquisition and Analysis (*35*) (Fig. S2).

### Statistical analysis

Statistical analysis and graphing were done using OriginPro (*36*). The values presented in the figures and tables of this work correspond to mean values±standard deviation. Mean values were compared using Student’s *t*-test, with the aid of Microsoft Excel analysis tools; p-values lower than 0.05 were interpreted as indicating a significant difference.

## RESULTS AND DISCUSSION

Pre-immobilization and post-immobilization bioimprinting were done with oleic acid and CTAB. Methanol, ethanol, *t*-butanol, *n*-heptane and toluene were also tested as bioimprinting agents since previous studies have shown that prior incubation in these solvents enhances the activity of free LipC12 (*28*, *30*).

### Pre-immobilization bioimprinting

Two pre-immobilization bioimprinting strategies were tested. In one strategy, purified LipC12 preparations were bioimprinted with the bioimprinting agents listed in Table 1 and then immobilized on Immobead 150P; this will be referred to as the “pre-pure” strategy. In the other strategy, crude cell-free extracts containing LipC12 were bioimprinted with the same bioimprinting agents and then immobilized on Immobead 150P; this will be referred to as the “pre-crude” strategy.

**Fig. 1:**
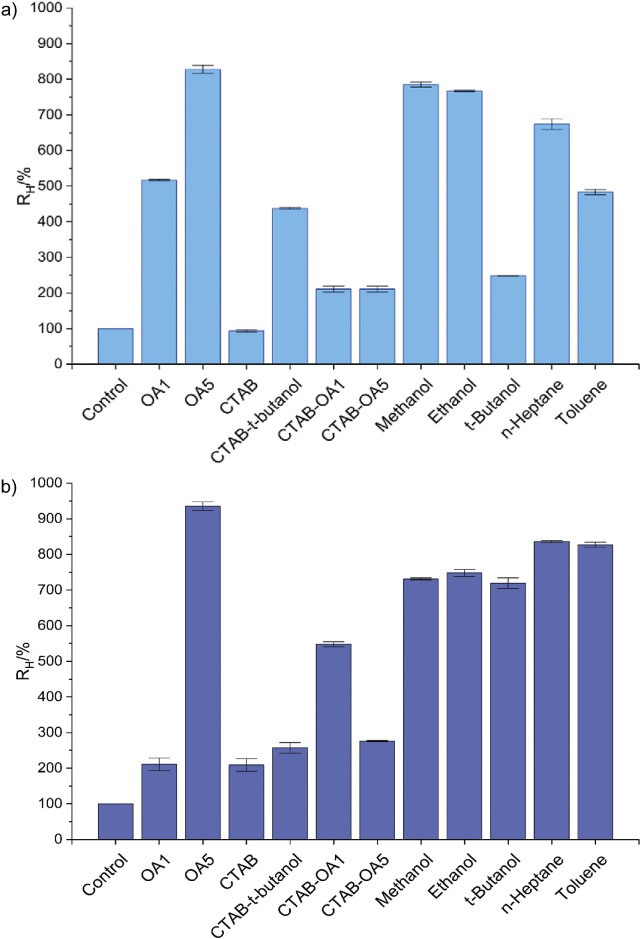
Relative triolein-hydrolyzing-activities (R_H_) in *n*-hexane obtained with the pre-immobilization bioimprinting of LipC12: a) results for LipC12 immobilized from a purified solution, b) results for LipC12 immobilized from the cell-free crude extract. The method for determination of triolein-hydrolytic activity in *n*-hexane and the calculation of the R_H_ value are described in section *Standard procedure for immobilization of LipC12*. The non-bioimprinted control (R_H_=100 %) had an activity of 50 U/g. The error bars represent the mean value±standard deviation (*N*=2). OA1 and OA5=oleic acid, *n*=29.4 and 147 nmol, respectively, CTAB=cetyltrimethylammonium bromide

With the pre-pure strategy, the most effective bioimprinting agent was OA5 (R_H_=828 %) (Fig. 1a). The next best bioimprinting agents were hydrophilic solvents, with R_H_ values of 785 % for methanol and 767 % for ethanol. Good results were also obtained with *n*-heptane (R_H_=673 %).

With the pre-crude strategy, again, the most effective bioimprinting agent was OA5 (R_H_=936 %) (Fig. 1b). The hydrophobic solvents gave the next best results, with R_H_ values of 837 % for *n*-heptane and 828 % for toluene. Good results were also obtained with the hydrophilic solvents, with R_H_ ranging from 720 to 748 %.

Although good results were obtained with OA5, treatment with OA1 (in which the oleic acid amount is a fifth of that in OA5) gave significantly lower R_H_ values, 235 % for the pre-crude strategy and 530 % for the pre-pure strategy. Comparing these two R_H_ values obtained with OA1, the lower value obtained for the pre-crude strategy might be due to adsorption of a significant proportion of the limited amount of oleic acid on the non-LipC12 proteins in the crude extract.

For all pre-immobilization bioimprinting treatments, the immobilization efficiencies (IE) were 100 % (Table S1), indicating that the presence of bioimprinting agents did not affect the immobilization of LipC12. All treatments showed higher activity retention values than the control (AR=204 % for the pre-pure strategy and AR=261 % for the pre-crude strategy), suggesting activation of LipC12 through the bioimprinting treatment (Table S2). Note that the AR values are directly proportional to the R_H_ values, as they are calculated based on the theoretical activity of LipC12 on the support and the measured activity of the immobilized preparation (see Eq. 2).

### Post-immobilization bioimprinting

Two post-immobilization bioimprinting strategies were tested. In one strategy, purified LipC12 was immobilized on Immobead 150P and the immobilized preparation was then bioimprinted with the agents listed in Table 1; this will be referred to as the “post-pure” strategy. In the other strategy, a crude cell-free extract containing LipC12 was immobilized on Immobead 150P and the immobilized preparation was then bioimprinted with the same bioimprinting agents; this will be referred to as the “post-crude” strategy. For both strategies (post-pure and post-crude), the immobilization efficiency (IE) was 100 % (Table S1). The AR values were 260 % for the post-pure strategy and 261 % for the post-crude strategy (Table S2).

**Fig. 2:**
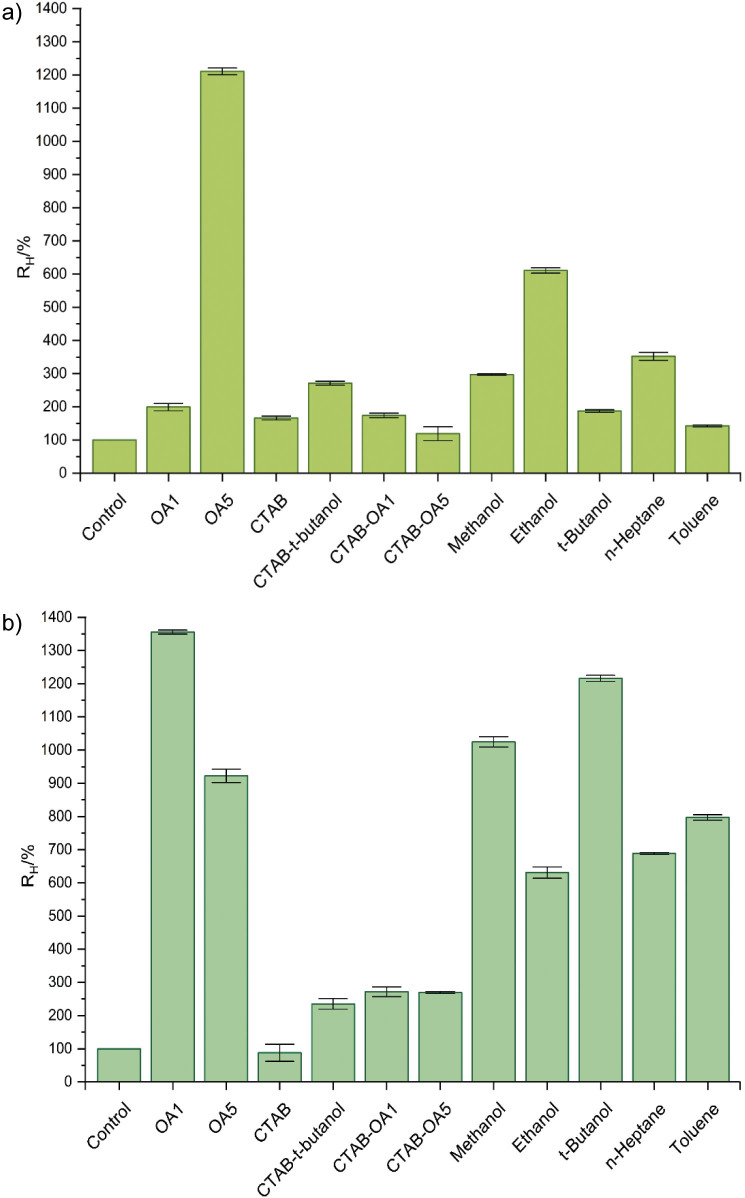
Relative triolein-hydrolyzing-activities (R_H_) in *n*-hexane obtained with the post-immobilization bioimprinting of LipC12: a) results for LipC12 immobilized from a purified solution, b) results for LipC12 immobilized from the cell-free crude extract. The method for determination of triolein-hydrolytic activity in *n*-hexane and the calculation of the R_H_ value are described in section *Standard procedure for immobilization of LipC12*. The non-bioimprinted control (R_H_=100 %) had an activity of 50 U/g. The error bars represent the mean value±standard deviation (*N*=2). OA1 and OA5=oleic acid, *n*=29.4 and 147 nmol, respectively, CTAB=cetyltrimethylammonium bromide

With the post-pure strategy, the most effective bioimprinting agent again was OA5 (R_H_=1211 %). The next best results were obtained with ethanol (R_H_=611 %) and *n*-heptane (R_H_=352 %) (Fig. 2a). With OA1, the R_H_ value was only 200 %.

With the post-crude strategy, the most effective bioimprinting agent was OA1 (R_H_=1356 %) (Fig. 2b). Two hydrophilic solvents gave the next best results, *t*-butanol (R_H_=1216 %) and methanol (R_H_=1024 %). With OA5, the R_H_ value was 923 %. With the solvents methanol, ethanol, *t*-butanol, *n*-heptane and toluene, there was a tendency for the R_H_ values obtained in the post-crude strategy (ranging from 600 to 1200 %) to be higher than the corresponding values obtained in the post-pure strategy (ranging from 100 to 600 %).

### The effects of solvents in pre-immobilization and post-immobilization bioimprinting

In our experiments reported above, both hydrophilic solvents (methanol, with logarithm of partitition coefficient log P=-0.77; ethanol, log P=-0.31; *t*-butanol, log P=0.57) and hydrophobic solvents (toluene, log P=2.73; *n*-heptane, log P=4.66) activated immobilized LipC12. However, there is no clear correlation between the degree of activation and the log P values of the solvents. Hydrophobic solvents typically interact with the lid domain of lipases, promoting its opening and enhancing catalysis, similar to the phenomenon of interfacial activation (*6*). The mechanism of activation of lipases by pretreatment with polar solvents is less clear, but the activation of LipC12 by methanol and ethanol is not surprising, as prior work showed that free LipC12 was significantly activated by preincubation (for 48 h at 4 °C) in 30 % aqueous solutions of methanol and ethanol, with relative activities of 1561 and 588 % respectively (*28*).

Some insight into the effects of solvents is given by the work of Liu *et al*. (*6*), who pretreated immobilized *Pseudomonas cepacia* lipase (PS) with pure organic solvents of varying log P values, molecular structures, and functional groups. These treatments induced changes in the secondary structure of PS: immobilized PS pretreated with all solvents had decreased contents of α-helices and β-turns and increased contents of β-sheets and random coils, regardless of whether the solvent activated the enzyme or not. However, it should be noted that Liu *et al*. (*6*) used pure organic solvents for the pretreatment, whereas we used around 20 % solvent in Tris-HCl buffer.

### Esterification activity of bioimprinted LipC12

In the bioimprinting experiments above, the relative activity of immobilized LipC12 (R_H_) was evaluated based on the hydrolysis of triolein in *n*-hexane. We selected the bioimprinting agents that performed best in this hydrolysis study (*i.e.* OA1 and OA5) and used them to evaluate the effect of bioimprinting on the esterification activity, namely the esterification of oleic acid with ethanol, in *n*-hexane.

**Fig. S3:**
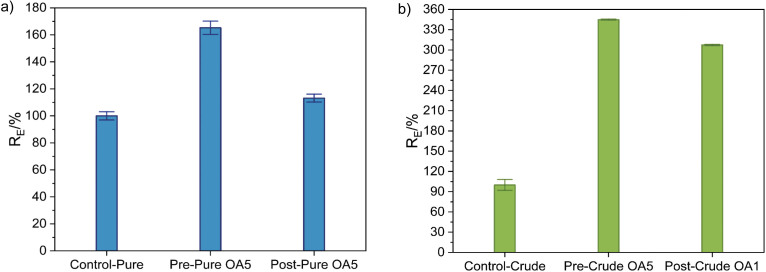
Relative esterification activities (R_E_) obtained with the best-performing bioimprinted LipC12 preparations: a) LipC12 immobilized from a purified solution, b) LipC12 immobilized from the cell-free crude extract. The method for determination of oleic acid esterification activity in *n*-hexane is described in section *Analytical ethods*. The error bars represent the mean value±standard deviation (*N*=3). OA1 and OA5=oleic acid, *n*=29.4 and 147 nmol, respectively

For both pre-immobilization bioimprinting and post-immobilization bioimprinting, the relative esterification activity (R_E_) was higher when LipC12 was immobilized from the crude extract than when it was immobilized from a purified preparation (Fig. S3). The best result, R_E_=345 %, was obtained with the pre-crude strategy, with OA5 as the bioimprinting agent. The next best result, R_E_=308 %, was obtained with the post-crude strategy, with OA1 as the bioimprinting agent. The low R_E_ values obtained when LipC12 was immobilized from the purified preparation contrast with the high R_H_ values obtained with these same preparations in the previous experiments. The fact that the bioimprinting was done with one of the products of triolein hydrolysis, namely oleic acid, might have contributed to the high R_H_ values. In the case of the ester synthesis reaction R_E_, it was not possible to investigate bioimprinting with the product (*i.e.* the ester), as the bioimprinting is done in aqueous medium and the lipase would hydrolyze the ester.

### Reuse of bioimprinted immobilized LipC12 in the hydrolysis of triolein in n-hexane

**Fig. 3:**
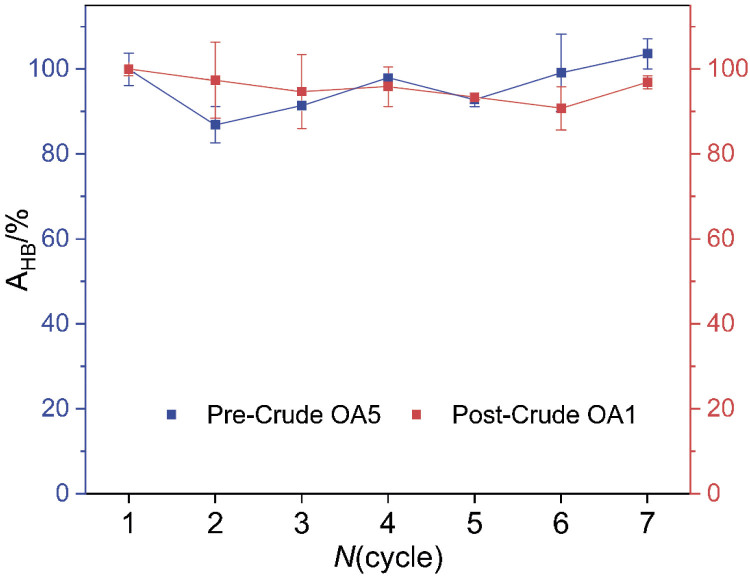
Reuse of bioimprinted LipC12 in the hydrolysis of triolein (A_HB_) in organic medium over seven reaction cycles. The method for determination of triolein-hydrolyzing activity in *n*-hexane and the calculation of the R_H_ value are described in section *Standard procedure for immobilization of LipC12*. The error bars represent the mean value±standard deviation (*N*=2). OA1 and OA5=oleic acid, *n*=29.4 and 147 nmol, respectively

Since the best results for triolein hydrolysis were obtained using LipC12 immobilized from the crude extract, for both pre-immobilization bioimprinting (R_H_=936 % for OA5) and post-immobilization bioimprinting (R_H_=1356 % for OA1), these bioimprinted immobilized preparations were evaluated for their reusability in successive cycles of triolein hydrolysis in *n*-hexane. Although there was experimental error of the order of ±10 %, both preparations retained essentially the same activity over seven reaction cycles (Fig. 3). In other words, the increased activity conferred by bioimprinting was maintained throughout the reuse cycles.

### Comparison with previous results for bioimprinting of immobilized lipases

Although there is a significant body of work concerning bioimprinting of immobilized lipases, our study is the first that compares pre-immobilization bioimprinting and post-immobilization bioimprinting. Previous works have used either pre-immobilization bioimprinting, or post-immobilization bioimprinting, or “simultaneous immobilization and bioimprinting” (in which the bioimprinting agent is added during the immobilization step itself). Further, our study is the first to compare bioimprinting of a lipase immobilized from a pure preparation with that of a lipase immobilized from a crude extract. Our results from this part of the study, in which we obtained the best relative hydrolytic activity with the post-crude strategy (R_H_=1356 %, for OA1), are especially encouraging, since immobilization from the crude extract avoids the costs of purifying the enzyme prior to immobilization.

Table 2 (*7*, *8*, *22*, *23*, *25*-*27*, *37*–*41*) shows previous studies in which immobilized lipases have been bioimprinted with fatty acids, with the results being evaluated in hydrolysis and esterification reactions. In this table, relative activities are defined in a manner analogous to Eq. 4, with the activity of the bioimprinted preparation being divided by that of the immobilized preparation without bioimprinting and then multiplied by 100 to give a percentage. When the results of relative activities were not provided by the authors, they were calculated from the activity values given in the paper. Most authors have used commercial enzymes. Also, most authors have immobilized the lipases by adsorption (Table 2), using either hydrophobic supports, such as Accurel MP-1000 (*25*) and NKA resin (*38*), or hydrophilic supports, like the ion exchange resin D152H (*27*). Bioimprinting prior to immobilization is the most used strategy. However, some authors have added bioimprinting agents during the immobilization itself, especially when immobilizing by encapsulation or producing crosslinked enzyme aggregates (CLEAs) (*7*, *8*, *13*, *37*).

**Table 2:** Best literature results for bioimprinting with fatty acids and its effect on hydrolysis and esterification reactions catalyzed by immobilized lipases

**Bioimprinting agent**	**Lipase source**	**Bioimprinting type/** ***t*(incubation)/min**	**Support/immobilization method**	**Substrate/solvent for activity determination**	**Relative activity^1^/%** **(Effect)**	***t*(reaction)/min**	**Reference**
Hydrolysis reaction							
Oleic acid in *t*-butanol	LipC12 (crude extract)	Pre-treatment/15	Immobead 150P/covalent bonding	Triolein/*n*-hexane	936	25	This work
		Post-treatment/15		Triolein/*n*-hexane	1356	25	This work
Oleic acid in *t*-butanol	LipC12 (purified fraction)	Pre-treatment/60	Immobead 150P/covalent bonding	Soybean oil/*n*-heptane	100	300	(*25*)
Oleic acid, Tween 60 in ethanol	*Candida rugosa* (CRL)	Pre-treatment/30	CLEA^2^	Fish oil/water	ND^3 ^(10.4 times higher hydrolysis degree)	15	(*7*)
Palmitic acid in PEG 400	*Geotrichum* sp.	Pre-treatment/ NI^4^	CLEA^2^	Fish oil waste/water	ND^3^ (higher hydrolysis degree)	480	(*37*)
Esterification reaction							
Oleic acid in *t*-butanol	LipC12 (crude extract)	Pre-treatment/15	Immobead 150P/covalent bonding	Oleic acid and ethanol/*n*-hexane	345	120	This work
Oleic acid in *t*-butanol		Post-treatment/15	Immobead 150P/covalent bonding	Oleic acid and ethanol/*n*-hexane	308	120	This work
Oleic acid in 1-pentanol	LipC12 (purified fraction)	Pre-treatment/60	Immobead 150P/covalent bonding	Oleic acid and 1-pentanol/*n*-heptane	128	300	(*25*)
Oleic acid in isopropanol	*Burkholderia cepacia* (BCL)	Pre-treatment/20	Dendrimer-functionalized magnetic nanocomposite/ covalent bonding	1-dodecanol and lauric acid/*i*-octane	110	30	(*23*)
Decanoic acid in acetone	*Burkholderia cepacia* (BCL)	Pre-treatment/60	CLEA^2^	Lauric acid and *n*-octanol/*i*-octane	154	120	(*8*)
Lauric acid, sorbitol, sucrose and lecithin	*Rhizopus oryzae* (ROL)	Pre-treatment/10	Acrylic resin/adsorption	Lauric acid and laurinol/solvent-free	209	120	(*26*)
Lauric acid and *n*-heptane	*Yarrowia lipolytica*	Pre-treatment/60	Resin D152H/adsorption	Lauric acid and *n*-dodecanol/solvent-free	231	120	(*27*)
Lauric acid and *i*-propanol	*Burkholderia cepacia* (BCL)	Pre-treatment/60	NKA resin/adsorption	Oleic acid and ethanol/solvent-free	119	120	(*38*)
Lauric acid and silane precursors of the sol-gel matrix	*Burkholderia cepacia* (crude extract)	Simultaneous^5^/1460	Sol-gel matrix/entrapment	Lauric acid and lauryl alcohol/isooctane	348	N.I.	(*39*)
Oleic acid, *t*-butanol and methanol	*Geotrichum* sp.	Pre-treatment/10	Acrylic resin/adsorption	Oleic acid and methanol/*n*-hexane	ND^3^ (6 times higher esterification activity)	120	(*4*0)
Lauric acid and PEG	*Burkholderia cepacia* (BCL)	Simultaneous^5^/1460	Silica gel/ Entrapment	Lauric acid and lauryl alcohol/isooctane	350	180	(*41*)
Commercial blending of fatty acids and ethanol	*Candida rugosa* (CRL)	Post-treatment/20	Polypropylene powder/adsorption	Oleic acid and ethanol/buffer	168	120	(*22*)
Oleic acid in *n-*butanol	*Candida rugosa* (CRL)	Pre-treatment/20	CLEA^2^ in PTFE filter membrane	Oleic acid and *n*-butanol/isooctane	104	210	(*13*)

^1^The relative activity was calculated based on the immobilized preparation without bioimprinting. The values in the table are either provided by the authors or calculated from the results in the paper; ^2^CLEA=cross-linked enzyme aggregates; ^3^ND=not determined; the degree of hydrolysis with the bioimprinted CLEA was compared to the free lipase; ^4^NI=not informed; ^5^Simultaneous treatment: the bioimprinting agents were added simultaneously with the enzyme to the sol-gel matrix or during the production of CLEAs. PEG=polythylene glycol, PTFE=polytetrafluoroethylene

Our best relative hydrolytic activity, 1356 %, obtained with post-immobilization bioimprinting, is higher than the relative hydrolytic activities that have been reported previously for pre-immobilization bioimprinting and simultaneous immobilization and bioimprinting (Table 2). Notably, there have been no prior reports on hydrolytic activities obtained with post-immobilization bioimprinting. The most closely related study is that of Sánchez *et al*. (*25*). They bioimprinted LipC12 with oleic acid for 60 min prior to immobilization on various supports, including Immobead 150, Accurel MP 1000, polypropylene powder, Nanomer I.44P (a nanoclay containing approx. 40 % dimethyl dialkyl amine by mass), and chitosan. The immobilized preparations were utilized in both the hydrolysis of soybean oil and the esterification of oleic acid with 1-pentanol. The bioimprinting treatment significantly increased the conversions for almost all their immobilized preparations, with the most notable enhancements occurring in those derived from hydrophobic supports. However, Sanchez *et al*. (*25*) only reported conversions after 5 h and they did not report the relative initial activities of bioimprinted LipC12 as we did in the current work. Also, the reactions that they used to evaluate the effect of bioimprinting on hydrolysis and esterification are different from those that we used. These differences in the strategy for evaluating the effects of bioimprinting makes it difficult to compare our results directly with theirs. In any case, in their work, bioimprinted and non-bioimprinted LipC12 gave no difference in conversions for the hydrolysis of soybean oil (*i.e.* the relative conversion at 5 h was 100 %), whereas in our work post-immobilization bioimprinting gave a relative activity of 1356 %. Likewise, in their work, bioimprinted LipC12 gave a relative conversion at 5 h of 128 % for the esterification of oleic acid with 1-pentanol, whereas in our work post-immobilization bioimprinting gave a relative activity of 308 % for the esterification of oleic acid with ethanol. This comparison shows that post-immobilization bioimprinting is a good alternative for improving LipC12 activity.

For relative esterification activities (Table 2), our best value of 345 % for post-immobilization bioimprinting with the higher oleic acid amount (OA5) is among the highest reported in the literature, comparable to those obtained for silica gel entrapment (350 %) (*41*) and sol-gel matrix entrapment (348 %) (*39*), both involving simultaneous bioimprinting and immobilization onto CLEAs. The only study reporting results for post-immobilization bioimprinting was done with *Candida rugosa* lipase (CRL) immobilized on polypropylene powder (*22*), with the bioimprinting agents consisting of a low concentration of a commercial blend of fatty acids (C_14_-C_16_), ethanol, and buffer at pH=7.0, resulting in a relative esterification activity of 168 %.

Although our bioimprinting strategies enhanced LipC12 activity in the current work, we used a specific combination of lipase, substrates, immobilization support, bioimprinting agent, and activity assay. Further research is necessary to confirm whether these strategies are effective for a broad range of lipases.

## CONCLUSIONS

Our study represents the first comparison of the strategies of pre-immobilization and post-immobilization bioimprinting and also the first comparison of bioimprinting of enzymes immobilized from a pure enzyme preparation and from a crude cell-free extract. Our work shows that these strategies are potentially quite useful. Good triolein-hydrolyzing-activity in *n*-hexane and good ethyl-oleate-synthesizing activity in *n*-hexane were obtained with bioimprinting of the metagenomic lipase LipC12 immobilized from a crude extract, with oleic acid dissolved in *t*-butanol as the bioimprinting agent. Relative to non-bioimprinted controls, this strategy gave a 13.6-fold increase in triolein-hydrolyzing-activity and a 3.5-fold increase in ethyl-oleate-synthesizing activity. Bioimprinting of lipases immobilized from the crude extract is especially promising as it avoids the costs of purifying the enzyme prior to immobilization. Moreover, the reusability experiments of bioimprinted LipC12 showed that the activation of LipC12 by oleic acid was not lost over seven reaction cycles.

## Appendix

**Table S1:** Activities and protein concentrations of the supernatants containing LipC12, before and after immobilization, along with immobilization efficiency (IE)

**Sample/bioimprinting strategy**	Activity/(U/mg)		*γ*(protein)/(mg/mL)		IE*/%	IE**/%
	Initial	Final	Initial	Final		
**Pre-pure**	1883	0	1.0	0	100	100
**Pre-crude**	1914	0	20.0	4.0	100	80
**Post-pure**	1780	0	1.0	0	100	100
**Post-crude**	1934	0	20	3.8	100	81

*IE calculated from the residual activity after the immobilization, **IE calculated from the residual protein content after the immobilization

**Table S2:** Activity retention (AR) of immobilized LipC12 with different bioimprinting strategies

**Bioimprinting agent**	**AR/%**			
	Pre-pure	Pre-crude	Post-pure	Post-crude
**None (control)**	204	261	260	261
**OA1**	1348	552	520	3531
**OA5**	2158	2437	3154	2403
**CTAB**	246	547	432	228
**CTAB-*t*-butanol**	1141	671	705	612
**CTAB-OA1**	551	1429	455	708
**CTAB-OA5**	550	721	310	703
**Methanol**	2045	1904	774	2668
**Ethanol**	1997	1949	1593	1643
***t*-Butanol**	648	1875	489	3167
***n*-Heptane**	1754	2179	917	1793
**Toluene**	1259	2156	370	2076

OA1 and OA5=oleic acid, *n*=29.4 and 147 nmol, respectively, CTAB=cetyltrimethylammonium bromide
